# Vascular Endothelial Effects of Sacubitril/Valsartan in Heart Failure With Reduced Ejection Fraction: Randomized Controlled Trial

**DOI:** 10.1016/j.jacadv.2024.101392

**Published:** 2024-11-13

**Authors:** Matthias P. Nägele, Thomas Haider, Leonie Kreysing, Jens Barthelmes, Delia Nebunu, Valentina A. Rossi, Monika Hebeisen, Isabella Sudano, Frank Ruschitzka, Andreas J. Flammer

**Affiliations:** a Cardiology, University Heart Center Zurich, University Hospital Zurich, Zurich, Switzerland; b Department of Biostatistics, Epidemiology, Biostatistics and Prevention Institute, University of Zurich, Zurich, Switzerland

**Keywords:** endothelial dysfunction, flow-mediated vasodilation, heart failure with reduced ejection fraction, microvascular dysfunction, retinal vessel analysis, sacubitril/valsartan

## Abstract

**Background:**

The mechanism of how sacubitril/valsartan improves outcomes in heart failure with reduced ejection fraction (HFrEF) is still incompletely understood.

**Objectives:**

The aim of this trial was to delineate the effects of sacubitril/valsartan on endothelial function, retinal microvascular function, and arterial stiffness in HFrEF.

**Methods:**

This double-blind controlled trial randomized 79 stable HFrEF patients with NYHA class II-IV on guideline-recommended therapy (mean age: 59.4 ± 12 years, left ventricular ejection fraction: 30% ± 7%) to sacubitril/valsartan or valsartan alone for 3 months. The primary endpoint was flow-mediated vasodilation (FMD). Secondary outcomes included flicker-induced dilatation of retinal arterioles and venules (FIDv), retinal arteriovenous ratio, and surrogate markers of arterial stiffness (pulse wave velocity and augmentation index).

**Results:**

The primary outcome FMD did not significantly differ between sacubitril/valsartan and valsartan alone (FMD 6.6% ± 3.9% vs 6.7% ± 2.8%; ANCOVA coefficient adjusted for baseline values 0.36, 95% CI: −0.78 to 1.51, *P* = 0.53). The secondary outcomes flicker-induced dilatation of retinal arterioles, arteriovenous ratio, pulse wave velocity, and augmentation index showed no significant differences. FIDv was lower after sacubitril/valsartan versus valsartan alone (FIDv 2.4% ± 1.3% vs 3.1% ± 2.1%; ANCOVA coefficient −0.7, 95% CI: −1.4 to −0.02, *P* = 0.04). Systolic blood pressure was lower after sacubitril/valsartan versus valsartan alone (ANCOVA coefficient −6.5 mm Hg, 95% CI: −12.7 to −0.3 mm Hg, *P* = 0.04). There were numerically fewer serious adverse events with sacubitril/valsartan versus valsartan alone.

**Conclusions:**

In this randomized double-blind clinical trial addressing mechanisms, sacubitril/valsartan lowered blood pressure and flicker-induced dilatation of retinal venules in patients with symptomatic HFrEF but did not improve endothelial function, retinal microvascular function, or arterial stiffness compared to valsartan monotherapy. (Differential Vascular and Endocrine Effects of Valsartan/​Sacubitril in Heart Failure With Reduced Ejection Fraction [VASCEND]; NCT03168568)

Sacubitril/valsartan is a first-in-class angiotensin receptor neprilysin inhibitor that is approved for the treatment of heart failure with reduced ejection fraction (HFrEF).^1^ The drug consists of a 1:1 complex of the angiotensin receptor blocker valsartan and the neprilysin inhibitor sacubitril. In the PARADIGM-HF trial, sacubitril/valsartan was superior to the angiotensin converting enzyme (ACE)-inhibitor enalapril in reducing the risks of death and heart failure hospitalization in patients with HFrEF.^2^

The precise mechanism as to why combined angiotensin receptor and neprilysin blockade is superior to ACE inhibition or blockade of the angiotensin receptor alone is still unclear. Neprilysin is a membrane-bound metalloprotease that breaks down a wide range of peptide hormones, including atrial and brain natriuretic peptide (BNP), endothelin-1, angiotensin II, substance P, and bradykinin. Sacubitril/valsartan increases the levels of BNP, and accordingly higher levels of cyclic guanosine monophosphate, one of its intracellular second messengers, are seen in heart failure patients treated with the drug, which may explain part of the beneficial effects.^3^

Many of the peptides affected by neprilysin blockade also act on vascular endothelial cells, and detailed studies on the vascular effects of sacubitril/valsartan in humans are lacking so far. The defining feature of a healthy vasculature is a well-functioning and reactive endothelium with intact nitric oxide (NO) signaling and sufficient vasodilatation in response to hormonal triggers and shear-stress.^4^ Endothelial dysfunction is the state in which these processes are disturbed and vessels fail to dilatate appropriately in response to stimuli. Endothelial dysfunction is a hallmark of heart failure and correlates with symptoms and adverse outcome.^5^

Therefore, the goal of this trial was to study whether sacubitril/valsartan results in an incremental improvement of vascular and endothelial dysfunction as measured flow-mediated vasodilatation (FMD) over valsartan alone in patients with HFrEF (Central Illustration).

**Central Illustration:**
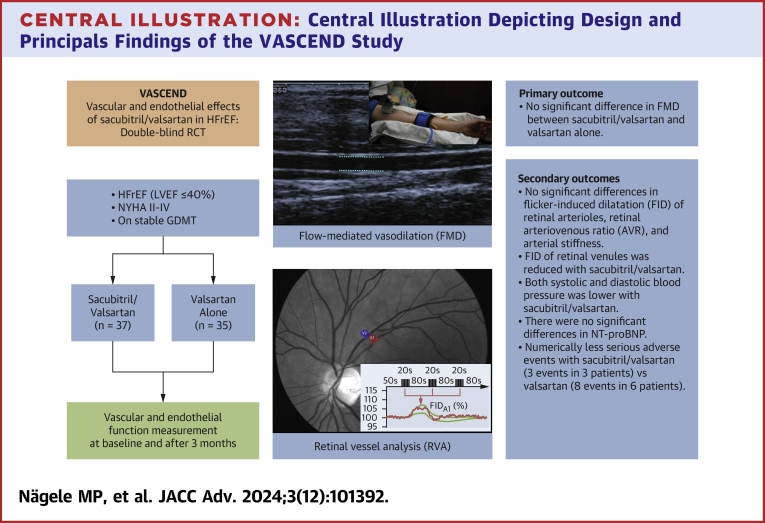
**Central Illustration Depicting Design and Principals Findings of the VASCEND Study** AIX = augmentation index; DBP = diastolic blood pressure; FIDa = flicker-induced dilatation of retinal arterioles; FIDv = flicker induced-dilatation of retinal venulse; FMD = flow-mediated vasodilation; GDMT = guideline-directed medical therapy; HFrEF = heart failure with reduced ejection fraction; PWV = pulse wave velocity; SAE = serious adverse events; SBP = systolic blood pressure.

## Material and methods

### Study design

This study was a single-center, double-blind, parallel 1:1 randomized controlled trial that compared the vascular effects of sacubitril/valsartan with valsartan in subjects with chronic heart failure with reduced ejection fraction. Patients were recruited at the Clinic of Cardiology at the University Hospital Zurich between 2017 and 2020. All patients signed informed consent before inclusion into the study. The study was performed according to the protocol, the current version of the World Medical Association Declaration of Helsinki, ICH-GCP guidelines, and the local legally applicable requirements. The CONSORT guidelines were followed during preparation of the manuscript. The study was approved by the local ethics committee (Basec-Nr. 2016-01946) and registered at the ClinicalTrials.gov database (NCT03168568). The trial was funded by a grant from Novartis and the University of Zurich. The authors vouch for the accuracy and completeness of the presented data, for the fidelity of the trial to the protocol, and for the accurate reporting of adverse events.

The inclusion criteria were as follows: Patients ≥18 years of age, diagnosis of symptomatic heart failure (NYHA functional class II-IV) per European Society of Cardiology heart failure guidelines, left ventricular ejection fraction ≤40% (any one measurement made within the past 12 months using echocardiography or MRI was acceptable), and established guideline-recommended therapy with an ACE-inhibitor, angiotensin receptor blocker, and a beta-blocker, as clinically indicated and tolerated, at stable doses for at least 3 weeks prior to inclusion.

Exclusion criteria were as follows: history of angioedema, sitting systolic blood pressure <90 mm Hg at visit 1 (screening) or visit 2 (randomization), concurrent or planned treatment with valsartan/sacubitril, any ACEI, any angiotensin receptor blocker or renin inhibitor during the study period, current acute decompensated HF, estimated GFR <20 mL/min/1.73 m^2^ as measured by the Chronic Kidney Disease Epidemiology Collaboration formula at visit 1 (screening) or visit 2 (randomization), serum potassium >5.5 mmol/L at visit 1 (screening) or visit 2 (randomization), acute coronary syndrome, stroke, transient ischemic attack, cardiac, carotid or other major CV surgery, percutaneous coronary intervention or carotid angioplasty within the 3 months prior to visit 1, coronary or carotid artery disease likely to require surgical or percutaneous intervention within the 3 months after visit 1, implantation of a cardiac resynchronization therapy device within 2 months prior visit 1 or intent to implant a cardiac resynchronization therapy device within next 3 months, history of heart transplant, on a transplant list or with ventricular assistance device, presence of any other disease with a life expectancy of <6 months, presence of significant endocrine diseases, including primary hyperparathyroidism, Cushing’s disease, adrenal insufficiency, pituitary tumors, primary hyperaldosteronism, manifest hyperthyroidism or genetic endocrine disorders, presence of active acute infectious diseases, inability to follow the procedures of the study, lack of safe contraception, participation in another study with investigational drugs or device within the 30 days preceding and during the study, narrow-angle glaucoma, epilepsy, cimino-shunt on both arms.

### Interventions

After screening and obtaining informed consent, participants were 1:1 randomized to 3 months of sacubitril/valsartan or valsartan alone. Randomization was performed with a computerized mechanism using the commercial SecuTrial System via Interactive Response Technology in blocks of 10. The SecuTrial System, which was managed by the independent Clinical Trial Center Zurich, ensured that the randomization list was concealed from the study team and investigators. Both patients and study staff (including the personnel measuring vascular function) were blinded to the randomization group until the end of the study.

The study drugs were uptitrated in 2-week intervals (Supplemental Figure 1) based on prior RAS blocker dosage (Supplemental Table 1) starting either from 50 mg sacubitril/valsartan twice daily or 40 mg valsartan twice daily up to the target dose of 200 mg or 160 mg twice daily, respectively. At baseline and the final study visit, clinical history and exam, phlebotomy, and assessment of vascular function (flow mediated vasodilation, retinal vessel analysis, and measurement of arterial stiffness) were conducted. Vascular measurements were performed in the morning in fasted state, after abstaining from nicotine and coffee for at least 24 hours. The laboratory parameters included hematogram, potassium, creatinine, and estimated glomerular filtration fraction calculated by Chronic Kidney Disease-Epidemiology Collaboration formula, high-sensitivity CRP and troponin T, N-terminal pro–B-type natriuretic peptide (NT-proBNP), glucose, and a full lipid status. Titration visits with vital signs and safety laboratory (serum creatinine and potassium) were performed 2 and 4 weeks after the baseline visit. Patients not tolerating a dose level (eg, significant hyperkalemia, thresholds according to the 2016 European Society of Cardiology Heart Failure Guidelines) ^6^ were reduced to the prior dose level and are allowed to finish the study, given they tolerate that lower dose and no adverse events occurred that would necessitate to stop the drug. In case of a switch from an ACEI to the study drugs, a 36-hour wash-out period was mandated.

### Flow-mediated vasodilation (primary endpoint)

FMD was performed based on established methods.^7^ Briefly, the arterial diameter of the brachial artery of the upper arm is measured and tracked continuously with a 10 MHz linear array ultrasound transducer (Siemens Acuson X300, Siemens AG) and specialized software (FMD-Studio). Measurement consists of a 1-minute baseline, a 5-minute occlusion and a 4-minute measurement, and recovery period. After a 1-minute baseline measurement, a blood pressure cuff is inflated 50 mm Hg above systolic pressure at the lower arm for 5 minutes. Then the blood pressure cuff is released and hyperemia occurred. The FMD outcome was plotted as the percent increase in diameter from the baseline period mean diameter to the maximum diameter after cuff release. For validation purposes of the measurement, the endothelial independent effect was measured using pharmacological dilatation of the brachial artery after glycerol trinitrate (GTN) (0.4 mg sublingual dose, Nitrolingual Spray, Pohl-Boskamp) and the brachial artery diameter and blood flow was recorded. The reproducibility of our laboratory was already published.^8^

### Retinal vessel analysis

Dynamic and static retinal vessel analysis (RVA) was performed as previously described using established protocols with an Imedos Dynamic Retinal Vessel Analyzer and proprietary software (Imedos).^7^ Briefly, after induction of mydriasis of a randomly selected eye with 0.5% tropicamide eyedrops, dynamic RVA was performed, which measures dilatation of retinal vessels after provocation with flicker light. A 1-1.5 mm long segment of a retinal arteriole and venule in the upper temporal fundus was selected, approximately 0.5 to 2 optic disc diameters away from the optic disc with automatic wall tracking of the vessel diameter. The acquisition included a 50 second baseline period followed by three flicker provocation periods (12.5 Hz flicker) with 20 seconds length, each time followed by a recovery period of 80 seconds. After acquisition, the three flicker periods were averaged and percent dilatation of the vessel from baseline are calculated (flicker-induced dilatation of retinal arterioles and venules, FIDa or FIDv).

Static RVA was performed using a monochromatic fundus image with measurement of all retinal arterioles and venules in the area 0.5 to 1 optic disc diameters away from the optic disc (VesselMap 2 software, Imedos). The software added up the diameters of all arteries and veins using a predefined formula,^9^ to calculate the central retinal artery equivalent (CRAE) and vein equivalent (CRVE). These values reflect a virtual diameter of the central retinal vein and artery. Both values are then used to calculate the arteriovenous ratio (AVR = CRAE/CRVE).

### Arterial stiffness

Arterial stiffness was measured as previously described using the SphygmoCor system (AtCor Medical).^7^ Pulse wave analysis was measured at the level of the radial artery and plotted as augmentation index, adjusted for a heart rate of 75 beats/min.^10^^,^^11^ Pulse wave velocity (PWV) was measured between the carotid and femoral artery with calculation of the pressure wave transit time using a foot-of-the-wave to foot-of-the-wave method obtained with a single high-fidelity applanation tonometer (Millar). Transit time between arterial sites is determined in relation to the R wave of the electrocardiogram and obtained by subtraction from the delays between electrocardiogram and both pulses.

### Statistical analysis

Descriptive statistics are reported as the mean ± SD or median (IQR) for continuous variables, as well as number and percentage of total for categorical variables. Descriptive group comparisons of vascular parameters are done by t tests.

The primary study outcome was the difference in FMD between sacubitril/valsartan and valsartan at the final study visit. This outcome was analyzed using multiple linear regression, considering treatment group as variable of interest and FMD value at baseline as covariate (ANCOVA). In a sensitivity analysis, missing FMD values at follow-up were imputed and the imputed datasets analyzed analogously. It is assumed that the missing data is missing at random. Multiple imputation with chained equations (number of multiple imputations = 50) was performed with the R package MICE. Missing values in other outcomes or baseline characteristics were not imputed and complete case analysis was done.

Sample size calculation was based on a previous study from our laboratory which tested endothelial function after 8-week treatment with the ACEI ramipril or placebo in patients with rheumatoid arthritis.^12^ In this study, a significant improvement of FMD of 1.15% was observed with ramipril treatment while there was no significant difference with placebo. Hence, for the primary endpoint, a difference in FMD between valsartan-sacubitril and valsartan is hypothesized at 1.15% with an SD of 1.5%. At a significance level of 5%, 36 patients per treatment group are needed to reach a statistical power of 90% at the superiority level. Including an excess of approximately 10% for dropouts, we planned to include 40 patients per treatment group (80 patients total).

The secondary (exploratory) outcomes FIDa and FIDv, retinal AVR, arterial stiffness as determined by pulse wave analysis and PWV, systolic and diastolic blood pressure, and NT-proBNP were analyzed in the same way as the primary outcome. Effects of lab values on endothelial function are analyzed with two multiple linear regression models with either FMD or AVR at follow-up as outcome and treatment group, FMD or AVR at baseline, and NT-proBNP at baseline as explanatory variables. Outcome variables are log-transformed before analysis if they deviate from a normal distribution. Normality was assessed by a quantile-quantile plot. Analyses were carried out with the intention to treat principle. R version 4.1.2 (R Core Team, 2021) was used for all calculations. A *P*-value of <0.05 was considered significant.

## Results

### Baseline characteristics

**Figure 1:**
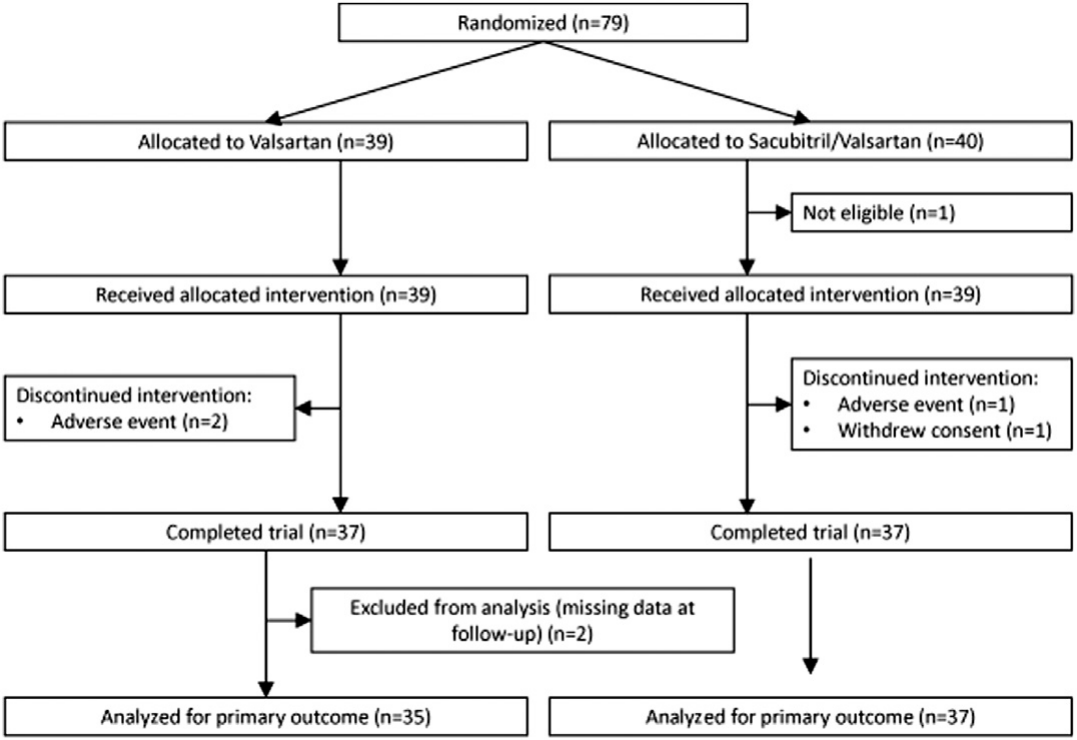
**Trial Flow Chart** Trial flow-chart of the VASCEND Study.

The trial flow chart is shown in Figure 1. After screening, 79 patients were randomized. One patient, who was initially randomized did not fulfil the inclusion and exclusion criteria before initiation of treatment and was excluded from the study leading to an intention-to-treat dataset of 78 patients. Two patients in the valsartan group stopped the study due to adverse events. One patient in the sacubitril/valsartan group stopped due to an adverse event and one patient withdrew consent. Two patients in the valsartan group had missing vascular assessments at the final study visit (FMD not measured due to technical reasons), resulting in 37 patients in the sacubitril/valsartan group and 35 patients in the valsartan group who finished the study assessments. At the final study visit, the mean daily doses in the sacubitril/valsartan and valsartan alone group were 254 ± 120 mg and 256 ± 86 mg, respectively.

The baseline parameters of the participants are shown in Table 1. The intervention groups were well balanced with respect to clinical characteristics, comorbidities, laboratory parameters, and drug therapy before randomization.

**Table 1:** Baseline Characteristics

	Overall (N = 78)	Valsartan (n = 39)	Sacubitril/Valsartan (n = 39)
Clinical characteristics			
Age (y)	59 ± 12	59 ± 13	60 ± 10
Female	6 (7.7)	1 (2.6)	5 (12.8)
Body mass index (kg/m^2^)	28 ± 4.7	28.7 ± 4.8	27.2 ± 4.6
Active smoking	23 (29.5)	9 (23.1)	14 (35.9)
Past smoking	40 (51.3)	22 (56.4)	18 (46.2)
Systolic BP (mm Hg)	122 ± 15	124 ± 15	120 ± 15
Diastolic BP (mm Hg)	73 ± 10	75 ± 11	71 ± 9
Heart rate (beats/min)	61 ± 9	61 ± 9	61 ± 9
Hypertension	49 (62.8)	25 (64.1)	24 (61.5)
Former stroke/MI	44 (56.4)	21 (53.8)	23 (59.0)
Dyslipidemia	55 (70.5)	27 (69.2)	28 (71.8)
Ischemic HF	53 (67.9)	25 (64.1)	28 (71.8)
LVEF (%)	30 ± 7	31 ± 6	29 ± 8
NYHA functional class			
II	69 (88.5)	35 (89.7)	34 (87.2)
III	8 (10.3)	3 (7.7)	5 (12.8)
IV	1 (1.3)	1 (2.6)	0 (0.0)
Laboratory parameters			
Hemoglobin (g/L)	142 ± 15	144 ± 15	139 ± 15
Leucocytes (g/L)	7.2 ± 1.8	7.2 ± 2.0	7.1 ± 1.6
eGFR CKD-EPI (ml/min/1.73 m^2^)	72 ± 19	75 ± 19	68 ± 19
CRP (mg/l), high sensitive	2.9 ± 3.5	3.0 ± 3.6	2.7 ± 3.3
Troponin T, high sensitive (ng/L)	19 ± 18	16 ± 12	21 ± 23
NT-proBNP (ng/L)	525 (234-1,330)	422 (184-1,137)	558 (338-1,657)
Glucose (mmol/L)	6.4 ± 1.7	6.5 ± 1.6	6.3 ± 1.8
LDL cholesterol (mmol/L)	2.1 ± 0.8	2.0 ± 0.9	2.1 ± 0.7
Triglycerides (mmol/L)	1.8 ± 1.0	1.7 ± 0.80	2.0 ± 1.1
Drug therapy before randomization			
ACE inhibitor	56 (71.8)	24 (61.5)	32 (82.1)
ARB	22 (28.2)	15 (38.5)	7 (17.9)
Beta-blocker	77 (98.7)	39 (100.0)	38 (97.4)
MRA	59 (75.6)	30 (76.9)	29 (74.4)
SGLT2 inhibitor	9 (11.5)	3 (7.7)	6 (15.4)
Loop diuretic	54 (69.2)	26 (66.7)	28 (71.8)
Metformin	13 (16.7)	4 (10.3)	9 (23.1)
Statin	60 (76.9)	32 (82.1)	28 (71.8)
Aspirin	39 (50.0)	18 (46.2)	21 (53.8)
Calcium-channel blocker	6 (7.7)	1 (2.6)	5 (12.8)

Values are mean ± SD, n (%), or median (IQR). Missing measurements (no. in brackets): Blood pressure and heart rate (4), LDL cholesterol (3), glucose (2).

ACE = angiotensin-converting enzyme; ARB = angiotensin receptor blocker; BP = blood pressure; CRP = C-reactive protein; eGFR CKD-EPI = estimated glomerular filtration fraction calculated using the chronic kidney disease-epidemiology collaboration formula; HF = heart failure; IQR = interquartile range; LDL = low-density lipoprotein; LVEF = left ventricular ejection fraction; MI = myocardial infarction; MRA = mineralocorticoid receptor antagonist; NT-proBNP = N-terminal pro–B-type natriuretic peptide; SGLT2 = sodium/glucose cotransporter 2.

### Primary outcome

**Figure 2:**
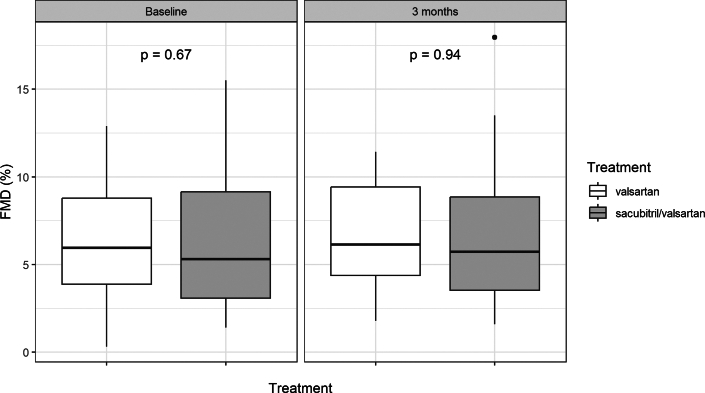
**Flow-mediated Vasodilation** Brachial artery flow-mediated vasodilation (FMD) at baseline and after 3 months of treatment with valsartan or sacubitril/valsartan. Box plots with interquartile range are shown.

The primary outcome of the study, FMD at follow-up corrected for baseline FMD, did not show a significant difference between sacubitril/valsartan and valsartan alone (ANCOVA coefficient 0.36, 95% CI [−0.78, 1.51], *P* = 0.53, Table 2). Similar results were obtained after multiple imputation of the six missing values (data not shown). The unadjusted analysis of FMD did not also show a significant difference between both groups (FMD at follow-up 6.6 ± 3.9% sacubitril/valsartan vs 6.7 ± 2.8% valsartan alone, *P* = 0.94; Table 3 and Figure 2). For validation purposes, brachial artery dilatation after GTN was measured (Tables 2 and 3). There were no significant differences in the GTN-mediated vasodilation between both study interventions (ANCOVA coefficient 1.46, 95% CI [−1.71, 4.63], *P* = 0.36).

**Table 2:** Analysis of Study Outcomes With ANCOVA Model

	Coef. Sacubitril/Valsartan	95% CI	*P* Value
FMD (%) (primary outcome)	0.363	−0.783 to 1.508	0.53
GTN (%)	1.458	−1.710 to 4.627	0.36
FIDa (%)	−0.112	−0.800 to 0.575	0.75
FIDv (%)	−0.713	−1.403 to −0.023	**0.04**
Retinal AVR	−0.006	−0.034 to 0.022	0.67
PWV (m/s)	−0.518	−1.530 to 0.494	0.31
AIX_HR75_ (%)	−2.082	−6.590 to 2.427	0.36
Systolic BP (mm Hg)	−6.545	−12.742 to −0.348	**0.04**
Diastolic BP (mm Hg)	−4.174	−7.544 to −0.804	**0.02**
NT-proBNP (μg/L)	0.035	−0.252 to 0.323	0.81
log NT-proBNP (μg/L)	−0.095	−0.390 to 0.201	0.52

Linear coefficient and its 95% confidence interval, treatment group comparison sacubitril/valsartan vs valsartan group analyzed using the same ANCOVA model as used for the primary study outcome (baseline parameter as covariate). Significant differences (*P*<0.05) are shown in **bold**.

AIX_HR75_ = augmentation index normalized to heart rate of 75/min; AVR = arteriovenous ratio; BP = blood pressure; FIDa = flicker-induced dilatation of retinal arterioles; FIDv = flicker-induced dilatation of retinal venules; FMD = flow-mediated vasodilation; GTN = glycerol trinitrate-mediated vasodilation; NT-proBNP = N-terminal pro–B-type natriuretic peptide; PWV = mean pulse wave velocity.

**Table 3:** Vascular Parameters at Baseline and Follow-up

	Valsartan (n = 39)	Sacubitril/Valsartan (n = 39)	*P* Value
Flow-mediated vasodilation			
FMD (%), baseline	6.39 ± 3.07	6.07 ± 3.62	0.67
FMD (%), follow-up	6.67 ± 2.75	6.60 ± 3.89	0.94
GTN (%), baseline	18.94 ± 7.73	17.45 ± 6.83	0.42
GTN (%), follow-up	17.57 ± 6.86	17.69 ± 6.64	0.95
Retinal vessel analysis			
FIDa (%), baseline	1.36 ± 1.83	1.32 ± 1.99	0.93
FIDa (%), follow-up	1.21 ± 1.88	1.12 ± 1.90	0.84
FIDv (%), baseline	3.32 ± 2.02	3.18 ± 1.34	0.72
FIDv (%), follow-up	3.08 ± 2.10	2.41 ± 1.28	0.11
Retinal AVR, baseline	0.86 ± 0.07	0.88 ± 0.06	0.39
Retinal AVR, follow-up	0.87 ± 0.09	0.88 ± 0.07	0.59
Arterial stiffness			
PWV (m/s), baseline	8.20 ± 2.31	7.75 ± 2.47	0.45
PWV (m/s), follow-up	7.92 ± 2.88	7.49 ± 2.20	0.50
AIX_HR75_ (%), baseline	21.83 ± 8.82	21.51 ± 11.19	0.89
AIX_HR75_ (%), follow-up	20.75 ± 9.47	18.08 ± 12.44	0.31
Blood pressure			
Systolic BP (mm Hg), follow-up	117 ± 16	110 ± 15	0.05
Diastolic BP (mm Hg), follow-up	71 ± 11	65 ± 7	**0.01**

Values are mean ± SD. Significant differences (*P*<0.05) are shown in **bold**.

AIX_HR75_ = augmentation index normalized to heart rate of 75/min; AVR = arteriovenous ratio; BP = blood pressure; FIDa = flicker-induced dilatation of retinal arterioles; FIDv = flicker-induced dilatation of retinal venules; GTN = glycerol trinitrate-mediated vasodilation; PWV = mean pulse wave velocity.

### Secondary outcomes

Analysis of secondary vascular outcomes are shown in Table 2 (ANCOVA model) and Table 3 (descriptive results). Due to technical reasons, between 2 and 13 patients had missing values in the intention to treat analysis of the secondary vascular outcomes and could not be considered in the respective analyses. Dynamic retinal vessel analysis showed no significant effect of sacubitril/valsartan on FIDa (ANCOVA coefficient −0.112, 95% CI: −0.800 to 0.575, *P* = 0.75). However, FIDv was reduced after sacubitril/valsartan compared to valsartan alone (ANCOVA coefficient −0.713, 95% CI: −1.403 to −0.023, *P* = 0.04). This difference was not significant in the unadjusted analysis (FIDv at follow-up 2.4% ± 1.3% sacubitril/valsartan vs 3.1 ± 2.1 valsartan, *P* = 0.11). No significant difference in retinal AVR was found between the groups (Tables 2 and 3).

Arterial stiffness, as determined by PWV, was not significantly different between sacubitril/valsartan and valsartan at follow-up (ANCOVA coefficient −0.518, 95% CI: −1.530 to 0.494, *P* = 0.31). Augmentation index, an indirect marker of vascular stiffness, did not show significant differences as well (ANCOVA coefficient −2.082, 95% CI: −6.590 to 2.427], *P* = 0.36).

Systolic and diastolic blood pressure (BP) was significantly reduced by sacubitril/valsartan compared to valsartan alone (ANCOVA coefficient for systolic BP −6.545, 95% CI: −12.742 to −0.348], *P* = 0.04 and for diastolic BP−4.174, 95% CI: −7.544 to −0.804], *P* = 0.02).

Regarding natriuretic peptides, NT-proBNP was not significant different between sacubitril/valsartan and valsartan alone (ANCOVA coefficient for log(NT-proBNP) −0.095, 95% CI: −0.390 to 0.201], *P* = 0.52). There was no correlation between the change in NT-proBNP (delta log (NT-proBNP)) and the change in the vascular outcomes throughout the trial (Supplemental Figure 2).

### Adverse events

The number of reported adverse events was 141 in total, 73 in sacubitril/valsartan, and 68 in valsartan treatment group. All adverse events are shown in Supplemental Table 2. Overall, the study treatments were well tolerated. Orthostatic dizziness including presyncope, syncope, and gait problems were reported more often under sacubitril/valsartan (23% of all adverse events) compared to valsartan alone (15% respectively). There were no numerical differences regarding hypotension and acute renal failure adverse events. Serious adverse events (shown in Supplemental Table 3) occurred numerically less often in patients randomized to sacubitril/valsartan (3 events in 3 patients) versus valsartan alone (8 events in 6 patients). The study drug was discontinued in 1 patient on sacubitril/valsartan and in 2 patients on valsartan due to adverse events. No deaths occurred during the trial.

## Discussion

In this randomized controlled trial addressing vascular mechanisms of sacubitril/valsartan in HFrEF, we observed no significant improvement in endothelial function as determined by FMD after 3 months of treatment with sacubitril/valsartan compared to valsartan alone. Secondary outcomes did not indicate a relevant effect on retinal microvascular function and arterial stiffness.

The underlying mechanism why combined angiotensin receptor and neprilysin inhibition is superior to angiotensin receptor or ACE blockade alone in HFrEF is still unclear. More insights on this question could help in the development of new therapies. Positive effects on other organs (that is improved glucose tolerance,^13^ preservation of kidney function^14^) support a systemic mechanism. As neprilysin cleaves a wide range of vasoactive peptides, a beneficial effect through the improvement of endothelial function seemed plausible.

Our study used FMD as the primary outcome, which is a well-established and noninvasive method for quantifying endothelial dysfunction. The lack of effect on FMD suggests that the benefits of sacubitril/valsartan are not mediated by an improvement of endothelial dysfunction. One explanation may be that neprilysin inhibition also increases concentrations of angiotensin II and endothelin I, which both counteract its benefits on NO thus resulting in a net neutral vascular effect.^15^ Similar results were observed in an animal study on rats with experimental HFrEF.^16^ The study revealed no significant difference in ex vivo endothelial function (relaxation of the thoracic aorta in response to acetylcholine) between sacubitril/valsartan and valsartan alone after 8 and 12 weeks of treatment. There was also no significant difference in plasma NO bioavailability and myocardial endothelial NO synthase expression. However, both myocardial NO bioavailability and myocardial expression of protein kinase G, a downstream effector of NO, was increased compared to valsartan alone, suggesting the beneficial effects were more confined to the myocardium rather than the peripheral vasculature or endothelium. With regard to human studies, our study was the first to investigate the effects of sacubitril/valsartan on endothelial function in a randomized and double-blind fashion. A previous study found an improvement of FMD after 3 months of sacubitril/valsartan in 11 patients with HFrEF; however, this study was open-label, uncontrolled, and unblinded.^17^ Another study in patients with HF and hypertension found an improvement in FMD after 6 months of sacubitril/valsartan compared to valsartan alone.^18^ However, this study was also not blinded, a less established FMD protocol was used, and concomitant drug treatment was not described.

With the lack of effect on endothelial dysfunction, our results may explain why sacubitril/valsartan does not reduce the risk for myocardial infarction and cardiovascular death in high-risk coronary artery disease patients compared to classical RAAS blockers.^19^^,^^20^ In these populations, endothelial dysfunction is a hallmark of the disease and most treatments that improve vascular outcomes, such as ACE inhibitors or statins, also improve endothelial dysfunction.^21^^,^^22^

Our study also measured retinal microvascular function as a secondary outcome. We have previously shown that both FIDa and FIDv are reduced in HFrEF compared to healthy controls.^7^ In this study, using the same flicker protocol, retinal microvascular function was impaired to a similar degree compared to our previously reported results. However, there was no improvement in FIDas after 3 months of treatment with sacubitril/valsartan. The reduced dilatation of retinal venules after sacubitril/valsartan is of unclear clinical significance. Most studies focused on the arterial microcirculation and less evidence is available for the role of venular flicker-induced dilatation in health and disease. The lack of effect on retinal AVR, a marker associated with long term risk of stroke,^23^ suggests that the relative vascular diameter and tone of retinal arterioles and venules were not affected by sacubitril/valsartan over the study period.

With regard to arterial stiffness, we did not find a benefit of sacubitril/valsartan on PWV, the gold standard for quantifying arterial stiffness, or augmentation index as determined by applanation tonometry. Similar results were seen in the animal study mentioned before, in which no significant difference in vascular compliance was seen between sacubitril/valsartan and valsartan.^16^ A recent small and uncontrolled study in patients with dilated cardiomyopathy with reduced EF did not show an improvement in arterial stiffness after 6 months of sacubitril/valsartan.^24^ Similar results were obtained in a randomized and double-blind study in patients with hypertension.^25^ Likewise, there was no effect on aortic impedance (a measure of central aortic stiffness) in the randomized EVALUATE-HF trial comparing sacubitril-valsartan with enalapril in patients with HFrEF.^26^ The stronger blood-pressure reducing effect of sacubitril/valsartan in our study is consistent with results from the PARAGON-HF trial, where mean systolic blood pressure was reduced more profoundly by sacubitril/valsartan (−4.5 mm Hg) as compared to valsartan alone.^27^

### Study Limitations

Although our study was powered for the primary endpoint, the limited sample size may have led to bias and a higher sample size may have been helpful to better identify smaller differences in the study outcomes. The study period of 3 months may have been too short to see effects on endothelial function. However, prior randomized studies indicate that 3 months is sufficient to induce relevant changes in cardiac biomarkers such as NT-proBNP when comparing sacubitril/valsartan to standard therapies.^28^ Unlike other studies, we did not observe a significant difference in NT-proBNP, possibly owing to the small sample size of the study. The lack of difference in NT-proBNP may partly explain the null finding of our study, as prior studies with significant endpoint differences (eg, PARADIGM-HF) also showed a significant parallel reduction in NT-proBNP. Due to the lack of differences in NT-proBNP, endothelial function and the sample size, we have opted against measurement of additional vasoactive peptides which may have helped to better understand the relationship between vasoactive peptides and endothelial function.

The achieved target doses of sacubitril/valsartan were lower in our study compared to the PARADIGM-HF trial, possibly due to the run-in phase in PARADIGM-HF, which excluded patients that failed to tolerate higher doses of the study drugs.^2^ By lacking a run-in phase, we do believe that our study is more reflective of the current clinical practice of how these drugs are used in HFrEF. The majority of participants were male, therefore generalization of the results to women is limited. Background treatment with statins and guideline-directed drugs for heart failure (eg beta-blockers or mineralocorticoid receptor antagonists) was high in our study. This may have attenuated any additional vascular benefits, as these agents are also known to improve endothelial function.^29^^,^^30^ The study was also not powered for its secondary outcomes. These outcomes should be regarded as exploratory and hypothesis generating. The original study plan also included exploratory measurement of additional endocrine hormones (ANP, BNP, oxytocin, renin, aldosterone and cortisol). This was not performed due to the lack of significant difference in the primary outcome as well as the secondary outcome NT-proBNP between the study groups.

## Conclusions

In this randomized trial addressing mechanisms, 3 months of treatment with sacubitril/valsartan compared to valsartan alone did not improve endothelial function, retinal microvascular function and arterial stiffness in patients with HFrEF. The effects of sacubitril/valsartan compared to valsartan on flicker-induced dilatation of retinal venules in patients with symptomatic heart failure set the stage for further investigating microcirculatory function in patients with heart failure.

Perspectives **COMPETENCY IN PATIENT CARE:** Our work informs clinicians that sacubitril/valsartan does not appear to mediate its benefit in HFrEF via the improvement of endothelial function. This sets the drug apart from other cardiovascular drugs such as ACE inhibitors or statins, which have been shown to improve endothelial function.

**TRANSLATIONAL OUTLOOK:** Future mechanistic studies on sacubitril/valsartan should focus more on the direct myocardial effects of the different vasoactive peptides that are increased by neprilysin inhibition. This may help in the development of new HF drugs.

## Funding support and author disclosures

This study was funded by 10.13039/100004336Novartis AG and the 10.13039/501100006447University of Zurich. The funders had no role in the design and conduct of the study, in the collection, analysis, and interpretation of the data, and in the preparation, review, or approval of the manuscript. Dr Nägele has received speaker fees by Vifor Pharma and AstraZeneca; congress travel support by 10.13039/100004319Pfizer; and consultancy fees from Boehringer Ingelheim and Pierre Fabre, all unrelated to this article. Dr Rossi has received congress travel support by 10.13039/100002429Amgen and 10.13039/100004326Bayer, unrelated to the article. Dr Barthelmes has received speaker fees by Imedos Systems; and congress fees by Servier, unrelated to the article. Dr Ruschitzka has not received personal payments by pharmaceutical companies or device manufacturers in the last 3 years (remuneration for the time spent in activities, such as participation as steering committee member of clinical trials and member of the Pfizer Research Award selection committee in Switzerland, were made directly to the University of Zurich). The Department of Cardiology of the University Hospital Zurich has received research-, educational- and/or travel grants from 10.13039/100000046Abbott, 10.13039/100020297Abiomed, 10.13039/100006396Alexion, 10.13039/100002429Amgen, AstraZeneca, At the Limits Ltd, 10.13039/100004326Bayer, Berlin Heart, B. Braun, 10.13039/100007497Biosense Webster, Biosensors Europe AG, 10.13039/501100005035Biotronik, 10.13039/100002491BMS, 10.13039/100001003Boehringer Ingelheim, 10.13039/100008497Boston Scientific, 10.13039/100030777Bracco, Cardinal Health Switzerland, 10.13039/100030895Concept Medical, Corteria, 10.13039/100008322CSL, 10.13039/501100022274Daiichi Sankyo, Diatools AG, 10.13039/100006520Edwards Lifesciences, FomF GmbH, Guidant Europe NV (BS), Hamilton Health Sciences, 10.13039/100001696IHF, 10.13039/501100013348Innosuisse, Johnson/Johnson, Kaneka Corporation, Kantar, 10.13039/100016492Kiniksa, Labormedizinisches Zentrum, 10.13039/501100023518MedAlliance, Medcon International, Medical Education Global Solutions, 10.13039/100004374Medtronic, 10.13039/501100018918MicroPort, Monocle, 10.13039/100007054MSD, 10.13039/100019138Mundipharma Medical Company, 10.13039/100004336Novartis, 10.13039/501100004191Novo Nordisk, 10.13039/501100024580Orion, 10.13039/100004319Pfizer, Quintiles Switzerland Sarl, RecorMedical, 10.13039/100016545Roche Diagnostics, Roche Pharma, Sahajanand IN, 10.13039/100004339Sanofi, Sarstedt AG, 10.13039/501100011725Servier, SIS Medical, Sorin CRM SAS, SSS International Clinical Research, Stromal, Terumo Deutschland, Trama Solutions, V-Wave, Vascular Medical, Vifor, Wissens Plus, ZOLL. These grants do not impact on the personal remuneration of Dr Ruschitzka. Dr Sudano has received consulting fees, travel grant and honoraria from Amgen, AstraZeneca, Daiichi Sankio, Medtronic, MSD, Novartis, Recordati, Sanofi and Servier, all unrelated to the present article. Dr Flammer has received fees from Alnylam, Amgen, AstraZeneca, Bayer, Boehringer Ingelheim, Bristol Myers Squibb, Fresenius, Imedos Systems, Medtronic, MSD, Mundipharma, Novartis, Pierre Fabre, Pfizer, Roche, Schwabe Pharma. Vifor, and Zoll, all unrelated to this article. All other authors have reported that they have no relationships relevant to the contents of this paper to disclose.
