# Supplementary material for: Vascular Endothelial Effects of Sacubitril/Valsartan in Heart Failure With Reduced Ejection Fraction: Randomized Controlled Trial
Source: JACC Adv. 2024 Nov 13;3(12):101392. doi: 10.1016/j.jacadv.2024.101392 (PMC11600776; doi:10.1016/j.jacadv.2024.101392)
Supplement: Supplemental material [file mmc1.pdf]

Supplementary Figures and Tables

Supplementary Figure 1: Study flow chart with gradual uptitration of the study drugs

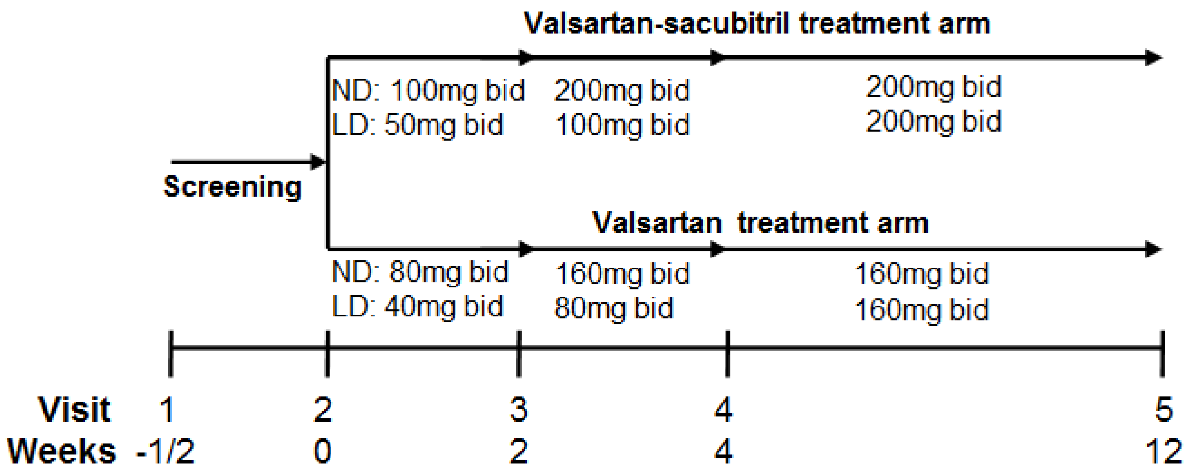

Abbreviations: ND, Normal dose titration group; LD, low dose titration group

Supplementary Figure 2: Plots of unadjusted analysis: delta of log of NT-proBNP versus delta of vascular parameters, between baseline and follow-up.

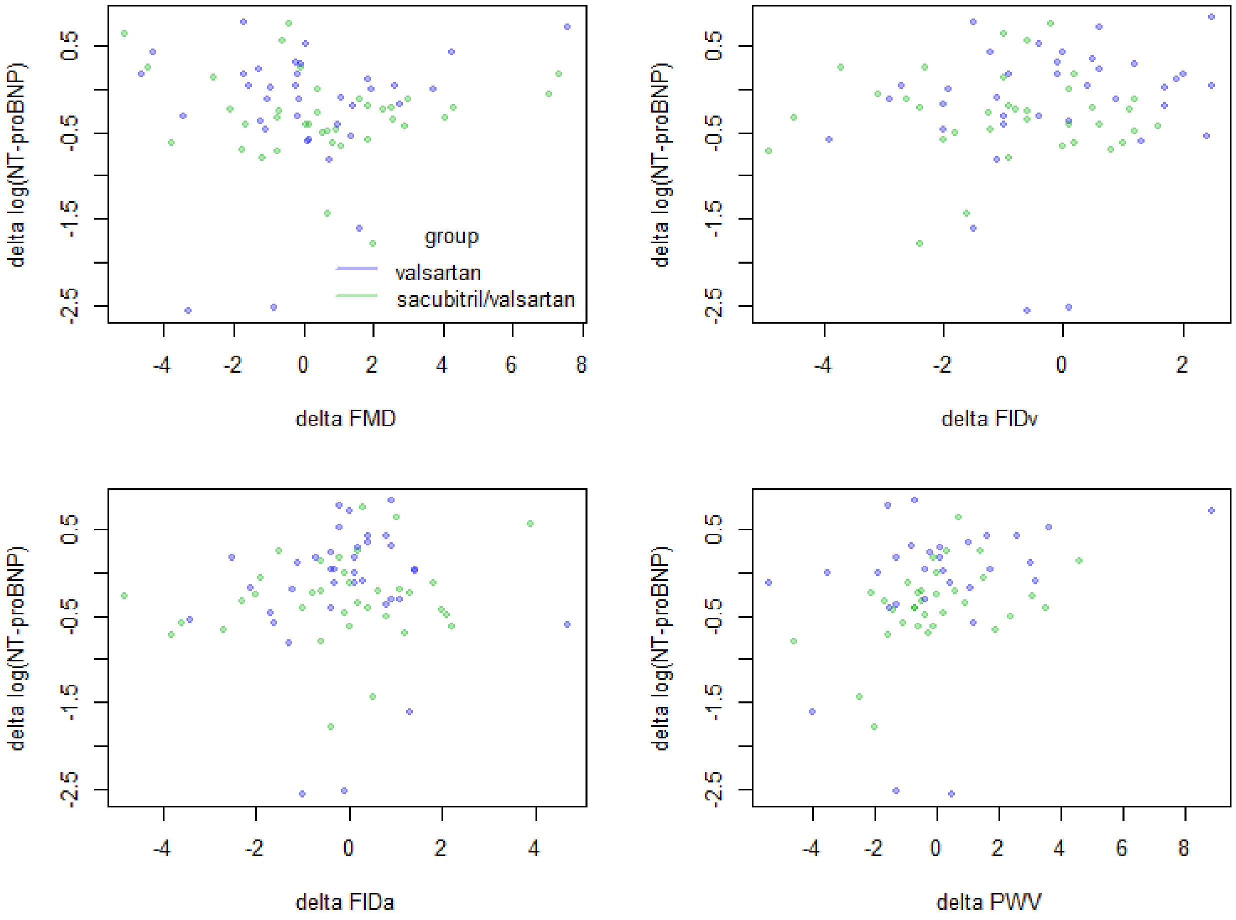

Supplementary Table 1: Minimum pre-study total daily doses of commonly used ACE-inhibitors and ARBs that enable patients to be dosed as the normal dose (ND) titration group.

ACE-inhibitor	Dose	ARB	Dose
Enalapril	10mg	Telmisartan	40mg
Captopril	100mg	Olmesartan	10mg
Lisinopril	10mg	Valsartan	160mg
Perindopril	4mg	Losartan	50mg
Ramipril	5mg	Irbesartan	150mg
Trandolapril	2mg	Eprosartan	400mg
Quinapril	20mg	Candesartan	16mg
Fosinopril	20mg		
Cilazapril	2.5mg		

Patients who had already been taking the maximum daily dose of Valsartan of 160mg b.i.d. were not uptitrated and directly received dose level 3 from visit 2 onwards. Patients were instructed to take their morning study drug doses at approximately 08:00 (8 AM) and their evening study drug dose at approximately 19:00 (7 PM).

Supplementary Table 2: All adverse events

	**Overall**	**Valsartan**	**Sacubitril/Valsartan**
**Total adverse events (n)**	141	68	73
**Diagnosis or main symptom (n, %)**			
(Orthostatic) dizziness, (pre)syncope, syncope, vertigo	27 (19.1)	10 (14.7)	17 (23.3)
Cough	10 (7.1)	4 (5.9)	6 (8.2)
Common cold, rhinitis, sore throat	9 (6.4)	4 (5.9)	5 (6.8)
headache	8 (5.7)	7 (10.3)	1 (1.4)
Tingling, paresthesia, pruritus	6 (4.3)	0 (0.0)	6 (8.2)
Flickering before the eyes, scintillating scotoma, impaired vision, feeling of sand in the eyes	5 (3.5)	3 (4.4)	2 (2.7)
Fatigue, lack of drive, loss of performance	5 (3.5)	4 (5.9)	1 (1.4)
Fever, Flu-like symptoms	4 (2.8)	3 (4.4)	1 (1.4)
Hypotension	4 (2.8)	2 (2.9)	2 (2.7)
Palpitations, tachycardia, rapid heart beat	4 (2.8)	2 (2.9)	2 (2.7)
Dyspnea, orthopnea, exertional dyspnea	3 (2.1)	1 (1.5)	2 (2.7)
Chest pain	3 (2.1)	1 (1.5)	2 (2.7)
Diarrhea	3 (2.1)	1 (1.5)	2 (2.7)
Weight gain	3 (2.1)	2 (2.9)	1 (1.4)
Cataract operation	3 (2.1)	2 (2.9)	1 (1.4)
Weakness, Fall	3 (2.1)	1 (1.5)	2 (2.7)
Nausea, Vomiting, Heartburn	3 (2.1)	0 (0.0)	3 (4.1)
Bloating, flatulence, constipation	3 (2.1)	0 (0.0)	3 (4.1)
Acute renal failure	2 (1.4)	2 (2.9)	0 (0.0)
ALT increase, increased creatinine	2 (1.4)	1 (1.5)	1 (1.4)
Hyperkalemia	0 (0.0)	0 (0.0)	0 (0.0)
Elective coronary angiography	2 (1.4)	1 (1.5)	1 (1.4)
Sweating	2 (1.4)	0 (0.0)	2 (2.7)
Abdominal pain	2 (1.4)	1 (1.5)	1 (1.4)
Tendovaginitis, onychia	2 (1.4)	1 (1.5)	1 (1.4)
Acute urinary retention	1 (0.7)	1 (1.5)	0 (0.0)
Atrial tachycardia (atrial fibrillation or atrial flutter)	1 (0.7)	1 (1.5)	0 (0.0)
Facial rash	1 (0.7)	0 (0.0)	1 (1.4)
Percutaneous angioplasty of the leg artery	1 (0.7)	1 (1.5)	0 (0.0)

Trouble falling asleep	1 (0.7)	1 (1.5)	0 (0.0)
Electrocardioversion due to atrial fibrillation	1 (0.7)	1 (1.5)	0 (0.0)
Endobronchial tumor in the lower lobe	1 (0.7)	1 (1.5)	0 (0.0)
Change in taste	1 (0.7)	0 (0.0)	1 (1.4)
Hypertensive crisis	1 (0.7)	1 (1.5)	0 (0.0)
Implantation of S-ICD	1 (0.7)	0 (0.0)	1 (1.4)
Derailment of INR (patient on vitamin K antagonist)	1 (0.7)	0 (0.0)	1 (1.4)
Feeling of cold	1 (0.7)	0 (0.0)	1 (1.4)
Left ventricular cardiac decompensation	1 (0.7)	1 (1.5)	0 (0.0)
Muscle cramps	1 (0.7)	1 (1.5)	0 (0.0)
Irritation of the ulnar nerve	1 (0.7)	0 (0.0)	1 (1.4)
Pulmonary vein isolation	1 (0.7)	1 (1.5)	0 (0.0)
Radiofrequency ablation	1 (0.7)	0 (0.0)	1 (1.4)
Hematoma	1 (0.7)	1 (1.5)	0 (0.0)
Pleuritis	1 (0.7)	0 (0.0)	1 (1.4)
Pain in the right groin	1 (0.7)	1 (1.5)	0 (0.0)
Contusion of the right knee	1 (0.7)	1 (1.5)	0 (0.0)
Subacromial impingement of left shoulder	1 (0.7)	1 (1.5)	0 (0.0)
Increased troponin	1 (0.7)	1 (1.5)	0 (0.0)

Supplementary Table 3: Serious adverse events

	**Overall**	**Valsartan**	**Sacubitril/Valsartan**
**Total serious adverse events (n)**	11	8	3
**Diagnosis or main symptom (n)**			
Palpitations, tachycardia, rapid heart beat	1	1	0
Dyspnea, orthopnea, exertional dyspnea	1	1	0
Acute renal failure	1	1	0
Elective coronary angiography	2	1	1
Endobronchial tumor in the lower lobe	1	1	0
Implantation of S-ICD	1	0	1
Left ventricular cardiac decompensation	1	1	0
Pulmonary vein isolation	1	1	0
Radiofrequency ablation	1	0	1
Subacromial impingement of left shoulder	1	1	0
**Causal relationship with study drug (at least possible causality) (n, %)**	3 (27.3)	3 (37.5)	0 (0.0)
